# The Effects of Maturation on the Colonic Microflora in Infancy and Childhood

**DOI:** 10.1155/2009/752401

**Published:** 2009-09-16

**Authors:** P. Enck, K. Zimmermann, K. Rusch, A. Schwiertz, S. Klosterhalfen, J. S. Frick

**Affiliations:** ^1^Department of Internal Medicine VI, University Hospital Tübingen, Frondsbergstrasse 23, 72076 Tübingen, Germany; ^2^Symbio Herborn Group GmbH, 35745 Herborn, Germany; ^3^Institute of Clinical Neurology and Medical Psychology, University of Düsseldorf, 40225 Düsseldorf, Germany; ^4^Institute for Medical Microbiology and Hygiene, University Hospital Tübingen, 72076 Tübingen, Germany

## Abstract

The composition of colonic mircoflora and its changes with maturation have rarely been investigated in large samples. *Methods.* We used conventional microbiological testing to analyse the colonic flora (Kyberstatus, Institut forMicroecology, Herborn, Germany) of stool samples from 12 484 children with different intestinal and nonintestinal diagnoses. Stool samples were analysed for total colony forming units (CFU) (per g stool) and the abundance of *Bifidobacteria, Bacteroides sp., Escherichia coli, Enterococcus sp.,* and *Lactobacillus sp.* with respect to age, gender. A subset of 1089 infants was analysed for monthly changes within the first year of life. *Results.* Total CFU and individual microbial species were highest during the first year of life, decreased within the first 2 years, and then stabilized for the remaining childhood. In infants, the total CFU rose until month 5, declined with weaning, and peaked at 9–10 months. Significant effects of age, but not of gender, were found in *Bacteroides sp.* and *Lactobacilli.* However *Bacterioids sp.* and *Lactobacilli* increased with age, while *Enterococci* and *E. coli* decreased, and Bifidobacteria remained stable. *Conclusion.* Colonic microflora show both a bacteria-specific and general pattern of maturation which is most profound within the first year.

## 1. Introduction

Age-related changes in the abundance of nonpathogenic bacteria found in the human colon have rarely been investigated in larger samples, neither in patients with intestinal and nonintestinal disorders nor in healthy subjects. In contrast, the effects of maturation on colonic flora in infants and children have occasionally been studied, but mostly in small cohorts of children.

Deviations in colonic flora have been shown to be responsible for respiratory diseases [1], intestinal diseases [2], and, especially, dermatologic disorders [3–6] in the pediatric population. It has, for example, been argued that the amount of exposure to commensal and pathogenic bacteria in the very early days of life, such as following a Cesarian section, [7] may contribute to the occurrence of atopic dermatitis later in life [8]. A similar argument is the basis for the “hygiene hypothesis” [9], which argues that greater exposure to environmental pathogens during childhood, for example, while growing up on a farm or having contact with animals, would protect for immunologically mediated diseases later in life [10, 11], but this is still a matter of debate [12]. However, the importance of commensal microbiota for the development of normal innate immunity is well established [13].

One reason for the relative absence of large-scale investigations is the suspicion that bacterial colonies found in stool samples would not remain stable during transport and storage; therefore. providing an inaccurate estimate of bacterial counts when conventional microbiological tools are used. Hence, commercial assays for fecal microbiota have not been a widely accepted diagnostic tool in routine clinical assessment. One exception is the use of fecal microbiota in the diagnosis of the inflammatory bowel diseases, especially Crohn's disease and ulcerative colitis, since these are strongly associated with *Clostridium diff*. colonization in both children [14] and adults [15].

Both the recent interest in prebiotic and probiotic treatment for functional intestinal [16, 17], other intestinal [18–21], and nonintestinal disorders [12–26], along with the availability of new molecular biological tools that are able to count different species, identify genetically different subspecies within each strain, and characterize normal human colonic microbiota and its variability, have created a new surge in research. Investigations regarding the effect of maturation on microbiota during childhood [27] and its change with aging [28] have recently been performed. However, as with most studies based on conventional microbiology, sample sizes have remained rather small thus far, as PCR technology is not yet easily available, and microarray chip technology that allows for the assessment of all bacterial species known to inhabit the human colon (in the range of >1000) is still very costly. This may, however, change in the near future.

The few studies that have assessed the effects of maturation on human fecal microbiota have shown that some bacterial species decline in their abundance with age, while other do not. This paper presents data from a large (>12,000 samples) conventional microbiological database of children with various intestinal and nonintestinal symptoms and diagnoses. Respective data regarding adult populations have recently been published [29]. The underlying hypothesis of this analysis is that the average bacterial abundance found in patients with various diseases and medical conditions may represent an approximation of what may be called the “normal” human fecal microbiota. Our assessment is based on conventional microbiological analyses that were performed in a commercial laboratory with GLP certification during the course of one year.

## 2. Material and Methods

### 2.1. Collection of Stool Samples for Microbiological Analysis

During the course of one year (2006), all fecal samples that were submitted by general practitioners for routine industrial microbiological analysis of nonpathogen colonic bacterial flora (Kyberstatus, Institute for Microecology, Herborn, Germany) were included in the study. In general, samples reached the laboratory within one day and were processed immediately. 

To ensure that the transport did not have any effect on the cultured species, a storage study was performed with 20 fresh samples. In short, 0.2 g of faeces was serially diluted in 1 mL of phosphate-buffered saline (PBS, pH 7.2). The solution was vortexed for 5 seconds and serially diluted (to 10^−9^) in PBS, pH 7.2. One mL of each dilution was plated onto enrichment or selective agar media.

The remaining feces were stored for three days at a temperature of 25°C, which represents the average temperature during shipment. Following the incubation period, the samples were processed as described and the results were compared. No significant discrepancy in the cell counts of the investigated microbiota could be detected within two days. Thus, it was concluded that a shipment of less than two days will have no effect on the composition of the cultivable microbiota. Only samples which arrived within one or two days of shipment were included in the study.

### 2.2. Identification and Enumeration of Microorganisms

Viable bacterial cell counts in feces were enumerated on the following selective media: Columbia blood agar (total cell count; BioMerieux, Nürtingen, Germany), U3G agar (enterobacteriacae, enterococci; Heipha, Heidelberg, Germany), Rogosa agar, (lactobacilli; Heipha), DIC agar (bifidobacteria; Heipha), Schaedler agar (bacteroides; Heipha), and SPM agar (clostridia; Heipha). Fecal samples were serially diluted in 1 mL of phosphate-buffered saline (PBS, pH 7.2) and subsequently plated on selective agar plates by a fully automated spiral plater capable of plating 12 agar plates simultaneously. Subsequently, the plates were incubated under either aerobic or anoxic conditions at 37°C for at least two days. Bacteria were first identified by Gram staining and colony morphologies. Additionally, identifications were performed by the API and VITEK systems (bioMérieux). All counts were recorded as the numbers of log_10_ CFU per mL of sample.

The following bacteria were routinely analyzed: *Clostridium sp.*, *Bifidobacteria*, *Bacteroides sp.*, subdominant (*E. coli*, *Enterococcus sp.*, *Lactobacillus sp.*), and other bacteria (*Pseudomonas sp.*, *Klebsiella sp.*, *Proteus sp.*, *Citrobacter sp.*, aerobic bacteria). Only bacteria that were identified in at least 50% of the respective subsamples (mentioned hereafter) were included into further analysis.

### 2.3. Additional Data

Since samples were from patients with various clinical diseases (Table 1), we collected additional data that was reported by the referring physician. This included age, gender, the presumed clinical diagnosis, and stool consistency and frequency. Stool pH was determined in the laboratory.

### 2.4. Data Analysis

After the data had been made anonymous, it was provided for further statistical analysis. Prior to the analysis, the sample was screened for identical patient IDs, and any second or subsequent analysis was excluded. Incomplete datasets were also excluded, except those where only gender information was missing.

**Figure 1:**
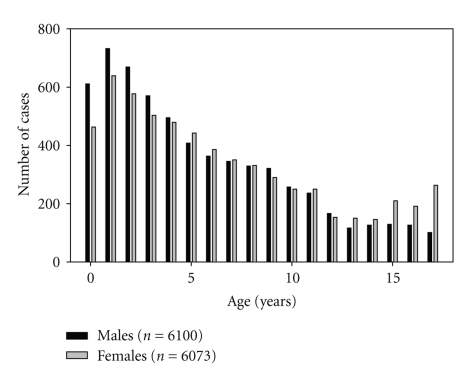
Age distribution (in years) in male and female subjects between 1 and 18 years.

The total age distribution allowed for the identification of a childhood sample (*n* = 12.484) (Figure 1) and an adult sample (*n* = 35.292). The adult study has been published recently [29]. A subset of 1089 infants (<1 year) was identified for the analysis of monthly changes within the first year of life.

Total CFU was analysed as well as the bacterial abundance for the following microbiota; *Bifidobacteria*, *Bacteroides sp.*, *lactobacilli*, *E. coli*, and *Enterococcus sp*. The percentage of subjects in which *Clostridium sp*. was identified was evaluated as well.

Prior to statistical analysis, data were normalized. The CFU of individual cell populations were compared to the total CFU identified by calculating the relative “abundance” of each bacterial species as ((specific CFU/total CFU)∗100).

The subsets (infants, children) were analyzed separately by ANOVAs for each bacterial count (abundance) with the between factors “age” (in monthly intervals for the infants, in yearly intervals for children) and “gender.” Post-hoc *t* tests with Bonferroni correction to account for multiple comparisons were used to test for single differences (between months and years, resp.) within each dataset. Pearson's *r* was computed to test for intercorrelation between single measures. All data are given as mean ± SEM. A threshold of 0.05 was set to indicate statistical significance in all tests. All data were analyzed using the SPSS Version 13 Statistical Package.

## 3. Results

From the sample of 47,775, *n* = 12483 were children and adolescents less than 18 years, and among them, *n* = 1089 were infants and babies less than one year of age. Their stool samples were analyzed for a variety of clinical conditions (see Table 1), but without any documentation of the diagnostic accuracy.

### 3.1. Infants

**Figure 2:**
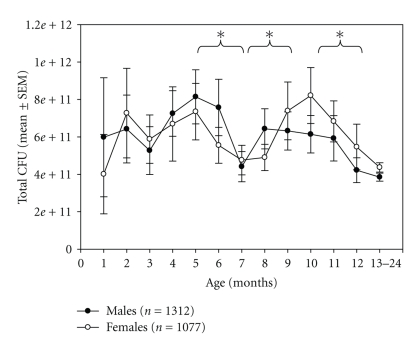
Total CFU (mean ± SEM) by age (months) and by gender. “∗” indicate significant difference in post-hoc *T*-test (Bonferroni corrected). A number of boys and girls are infants (604 : 459 m : f) plus children up to 2 years.

The infant group (≤1 year) included 604 males and 459 females, 6.47 ± 0.92 months of age. ANOVA revealed a significant effect of age (*F* = 3.94, *P* < 001), but not of gender on total CFU (Figure 2). As can be seen, CFU increased within the first few months, declined to a first low at the time of weaning, and exhibited a second peak at months 10 and 11. It steadily declined thereafter to reach stable levels at age 5 or 6 (described in what follows).

**Figure 3:**
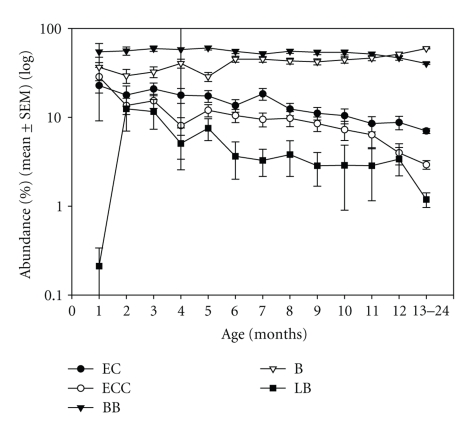
Abundance (%) (mean ± SEM) by age (months) of *E. coli*, *Enterococcus sp*., as well as L*actobacillus sp*., *Bifidobacteria*, and *Bacteroides sp*.

ANOVA showed significant effects of age, but not of gender on individual bacterial abundance. All bacteria except *Lactobacilli* were present within the first month of life. However, *Enterococci* and *E. coli* decreased (*F* = 3.098, *P* < .001 and *F* = 2.884, *P* = .001, resp.) while *Bacteroides sp.* increased (*F* = 3.14, *P* < .001), and *Bifidobacteria* remained stable (*F* = 1.226, *P* = .265) within the first year. *Lactobacilli* existed at high concentrations at month 2, but steadily decreased thereafter (*F* = 2.143, *P* = .016) (Figure 3). The percentage of *Clostridium sp*. declined from 3 to 2%.

### 3.2. Children

**Figure 4:**
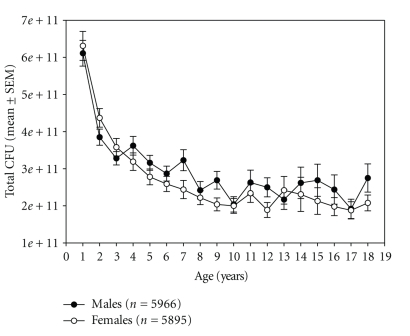
Total CFU (mean ± SEM) by age (years) and by gender.

For all the 12,483 children (6.02 ± 0.04 years, 6100 : 6074 male: female, remaining = missing), total CFU significantly declined (*F* = 126.74, *P* < .001) from year 1 to year 10 and remained stable thereafter. Overall, females had a significantly lower bacterial count than males (*F* = 19.24, *P* < .001) (Figure 4).

**Figure 5:**
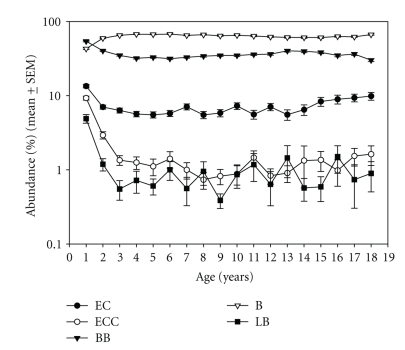
Abundance (%) (mean ± SEM) by age (years) of *E. coli*, *Enterococcus sp*., as well as L*actobacillus sp*., *Bifidobacteria*, and *Bacteroides sp*.

Individual bacteria abundance showed the same age-related decline within the first few years for *Enterococci* (*F* = 37.188, *P* < .001) and *Lactobacilli* (*F* = 9.888, *P* < .001), while *E. coli* moderately decreased (*F* = 8.679, *P* < .001). Given the high proportion of their specific CFU, the time profile of *Bifidobacteria* and *Bacteroides sp*. was almost complementary. *Bifidobacteria* decreased overall (*F* = 34.051, *P* < .001), while *Bacteroides sp*. increased their proportion (*F* = 43.465, *P* < .001) (Figure 5). An independent effect of gender was noted for *Bifidobacteria* (*F* = 4.982, *P* = .026) and *E. coli* (*F* = 12.203, *P* = .001) with higher values for *Bifidobacteria*, but lower values for *E. coli* in males (data not shown). The percentage of *Clostridium sp*. remained low at around 2% (data not shown).

**Figure 6:**
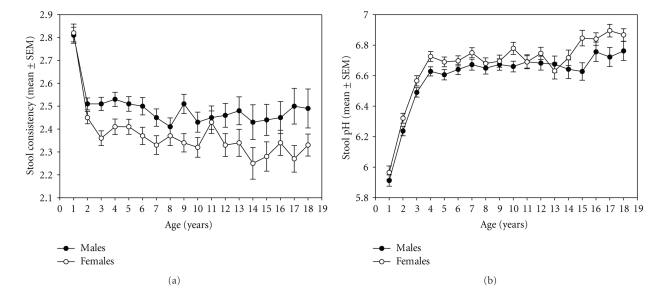
Stool consistency (mean ± SEM) (a) (1 = solid, 5 = liquid) and stool pH (mean ± SEM) (b) by gender and age (years).

Stool consistency (rated between 1 = solid and 5 = liquid) decreased (*F* = 16.108, *P* < .001) and was significantly lower in females (*F* = 51.735, *P* < .001). Stool pH increased within the first years (*F* = 74.425, *P* < .001) and was overall higher in females (*F* = 23.998, *P* < .001) (Figures 6(a) and 6(b)).

Moderate negative correlations were found between pH and *Bifidobacteria* (*r* = −.33, *P* < .001), and positive correlations between pH and *E. coli* (*r* = .24, *P* < .001).

## 4. Discussion

Newborns are germfree at the time birth, and acquire bacteria from their immediate environment within the first hours of life: from their mothers' vaginal and fecal flora, and from the hospital or home environment, depending on the mode and location of delivery. Other factors that can influence the composition of the intestinal microflora in newborns are the environment during birth, maturity, hygiene measures, and the type of infant feeding [30].

Specifically, *E. coli*, *Enterobacter sp.*, and *Enterococci* colonize the gastrointestinal tract rapidly, and are often found in higher concentrations in newborns than in adults due to the fact that aerobes and facultative anaerobes are favored by the intestinal milieu at birth. When these species expand, they consume oxygen and allow anaerobic strains to follow, including *Bacteroides*, *Bifidobacteria*, and *Clostridia*. *Lactobacilli* are oxygen tolerant and are most likely derived from the mothers' fecal flora [31]. Infants delivered by Cesarian section do not come into contact with the maternal flora during delivery. Their colonization of *Bacteroides sp*. is delayed. This has been shown to be responsible for the development of atopic dermatitis later in life [32].

However, among infants, the variability of the composition and profile of intestinal microbiota remains high [33]. It is also subject to change according to differences in nutrition [34] and other cultural influences [35], as well as host genetics, which exert a strong influence over the composition of the fecal microflora [36].

Therefore, the exploration of the “normal” range of the gut microbiota composition during the development and maturation remains a methodological challenge. In a previous paper [29], we reported the data from a large (>35.000 samples) adult cohort under the assumption that the average bacterial abundance across all patients may represent an approximation of what may be called the “normal” human fecal microbiota. Using the current data from the same source, we applied this argument to infants and children.

In agreement with previous reports (summarized by Adlerberths et al. [31]), we report a high total bacterial load in the newborn intestine within the first months after birth that declines within the first years of life to levels that are maintained during childhood and thereafter [29]. The total CFU may also reflect changes associated with weaning, since a local trough can be found (see Figure 2) at around age 7 months, which may reflect this change in nutrition. This seems somewhat counter-intuitive, as one would expect to find an increase in the CFU once more types of foods are introduced in the diet. However, weaning may induce an initial decline of predominantly lactate acid metabolizing bacteria while other species may follow colonizing the colon at a slower rate.

During the first year, the composition of the microbiota changes. *E. coli* and *Enterococci* decline, while *Bacteroides sp*. increases 3-folds. *Bifidobacteria* B. in the infant stool samples remained stable during the first years. Different from many previous reports [31], *Lactobacilli* were not present during the first month, but represented as much as 10% of the identified CFU one month later (Figure 3). Controversy in literature on whether or not *Lactobacilli* are part of the early gut colonizers has been noted [31], and it has been stated that the *Lactobacilli* colonizing the infant's gut are likely not from their mothers' vaginal flora [37], but rather from maternal gut flora. Colonization with *Lactobacilli* is delayed after Cesarian section, but reported to be normal one month later [38]. Since we do not know the route of birth in our sample, we cannot exclude that this may be the cause of the late occurrence of LB in our study. Other reasons postulated [31] for this discrepancy are differences in the methods used to identify *Lactobacilli*, or differences in the colonization pattern during the last decades, as many studies derive from work earlier than 1990.

During the remaining childhood up to age 10, the total CFU constantly declines to levels at about 2 × 10 E11 (see Figure 4), while individual strains reach stable levels at age 3 (see Figure 5). These findings also support previous reports [4, 31]. Similar to our adult sample [29], females had significantly lower stool consistency (towards more solid stools) and higher stool pH. Although both measures are not linked, they likely express the same function. We cannot exclude, however, that this may be due to differences in body weight and/or body mass. Unfortunately, the database did not allow adjusting for this.

Several other limitations of our analysis need to be addressed. One is that methods based on bacterial culture might not detect all species of the human microbiota. Additionally, obligate anaerobic bacteria might be at a disadvantage, and, therefore, the proportion of anaerobic bacteria to aerobic bacteria might not reflect the ratios in vivo. However, direct comparison of conventional culture analyses and molecular techniques have produced similar results, at least for the most abundant bacteria [39]. Other limitations refer to missing clinical data on medication intake, such as antibiotics. Nutritional habits and regular consumption of pre- and probiotic products may also have corroborated the results. For example, breastfeeding markedly influences the microbiotic flora in infants as does type of formula consumed and the time that supplementary food is introduced. Thus, we can only give an approximation of what may be called the ‘normal’ human faecal microbiota for this time period. Furthermore, the use of antibiotics severely influences intestinal flora. Since bacterial infection of the upper and lower respiratory tract is quite common, many children receive antibiotics during the first year of life. As these data were missing in our dataset, we cannot generalize our assumption. However, as stated in a previous paper [29], we reported the data from a large infant and child cohort (>12,000 samples) under the assumption that the average bacterial abundance across all patients may represent an approximation of what may be called the “normal” human fecal microbiota during infancy and childhood. 

## Figures and Tables

**Table 1:** Major diagnoses in children that initiated the stool analysis.

Organ classes	Main diagnoses	Children
Gastrointestinal	Unspecified	1862
	Irritable Bowel Syndrome	1188
	Crohn's disease	27
	Ulcerative colitis	64
	Diarrhea	771
	Constipation	296
	Candida	1061
	Food Intolerance	238

Respiratory	Unspecified	663
	Bronchitis	294
	Sinusitis	—
	Asthma	103

Urogenital	Unspecified	27
	Cystitis	—
	Genital mycosis	—

Dermatologic	Unspecified	40
	Dermatitis	256
	Psoriasis	28
	Acne	—

Allergic	Allergy, unspecified	229
	Neurodermititis	2184
	Recurrent urticaria	—
	Pollinosis	—
	Allergic asthma	230
	Food allergies	209
	Respiratory allergies	106

Rheumatologic	Arthritis	—

Others	Infection defence weakness	402
	Autoimmune diseases	—
	Malignom	—

Missing		421

Total		10699
		(85%)*

*The remaining to the total of 12.484 are cases with unknown diagnoses.
